# Maltose and Maltodextrin Utilization by *Listeria monocytogenes* Depend on an Inducible ABC Transporter which Is Repressed by Glucose

**DOI:** 10.1371/journal.pone.0010349

**Published:** 2010-04-27

**Authors:** Shubha Gopal, Daniela Berg, Nicole Hagen, Eva-Maria Schriefer, Regina Stoll, Werner Goebel, Jürgen Kreft

**Affiliations:** 1 Department of Studies in Microbiology, University of Mysore, Mysore, India; 2 Lehrstuhl für Mikrobiologie - Biozentrum, Universität Würzburg, Würzburg, Germany; 3 Institut für Pathologie, Technische Universität, München, Germany; 4 Lehrstuhl Bakteriologie, Max von Pettenkofer-Institut, Universität München, München, Germany; University of Hyderabad, India

## Abstract

**Background:**

In the environment as well as in the vertebrate intestine, *Listeriae* have access to complex carbohydrates like maltodextrins. Bacterial exploitation of such compounds requires specific uptake and utilization systems.

**Methodology/Principal Findings:**

We could show that *Listeria monocytogenes* and other *Listeria* species contain genes/gene products with high homology to the maltodextrin ABC transporter and utilization system of *B. subtilis*. Mutant construction and growth tests revealed that the *L. monocytogenes* gene cluster was required for the efficient utilization of maltodextrins as well as maltose. The gene for the ATP binding protein of the transporter was located distant from the cluster. Transcription analyses demonstrated that the system was induced by maltose/maltodextrins and repressed by glucose. Its induction was dependent on a LacI type transcriptional regulator. Repression by glucose was independent of the catabolite control protein CcpA, but was relieved in a mutant defective for Hpr kinase/phosphorylase.

**Conclusions/Significance:**

The data obtained show that in *L. monocytogenes* the uptake of maltodextrin and, in contrast to *B. subtilis*, also maltose is exclusively mediated by an ABC transporter. Furthermore, the results suggest that glucose repression of the uptake system possibly is by inducer exclusion, a mechanism not described so far in this organism.

## Introduction

*Listeriae* are gram-positive rods, asporogenic and very robust. They grow between pH 5 to 9, from 1–45°C and at salt concentrations up to 12%. The genus comprises eight species, *L. monocytogenes* and *L. ivanovii* are pathogenic for humans and/or animals, *L. seeligeri* is generally regarded as nonvirulent, the five species *L. innocua*, *L. welshimeri*, *L. grayi*, *L. marthii* and *L. rocourtiae* are harmless saprophytes. Natural habitats of *Listeriae* are decaying plant material in soil and also the intestine of healthy animals, including birds. The bacteria eventually gain access to sewage and water and may contaminate food processing environments. Uptake of contaminated food leads to the transmission of *Listeria* to humans [1], [2].

The multi-facetted systemic disease caused by the human-pathogenic species *L. monocytogenes*, listeriosis, is rare but has a high mortality in severe cases. It mainly occurs in children, pregnant, elderly and immunocompromised persons [3], [4]. The bacterium has also been implicated in a number of gastroenteritis cases [5].

*L. monocytogenes* is commonly regarded as model organisms for the important group of facultative intracellular bacterial pathogens [6], [3]. Also the occurrence and survival of *Listeria* in food processing environments and in food has intensively been studied [7], [8]. Much less is known about the occurrence of *Listeria* in soil [9], [10], [11] and in the environment [12], [13]. A few reports deal with the association of *Listeria* with plants [14], [15], [16]. Several factors governing the transition of pathogenic *Listeriae* from the saprophytic life in the environment to that of an intracellular pathogen have been reviewed recently [17], [18]. Bacterial mechanisms counteracting acid, salt and bile stress have been studied with respect to the survival in and the colonization of the intestinal tract [7], [19], [20]. However, nothing was known about the nutritional conditions of *Listeria* in this environment.

The bacterial habitat on decaying vegetation in soil is of enormous diversity and is ill-defined. But it can be assumed that it is rich in complex carbohydrates like starch and its degradation products maltodextrins and maltose. This also holds true for the intestinal tract of man and animals, the site of colonization for non-pathogenic and pathogenic *Listeria* species and invasion for pathogenic *Listeriae*. In the intestine, starch from food will be degraded to maltodextrins and maltose mainly by pancreatic amylase, independently of any amylolytic activity of colonizing bacteria. It has been shown that a number of bacteria found in the natural environment and also in the intestine dispose of efficient uptake mechanisms for maltodextrins/maltose. The paradigm for this is the maltodextrin system of *E. coli* [21], but related systems have also been found in gram-positives, for example in *Enterococcus faecalis* [22], *Staphylococcus xylosus* [23], *Streptococcus pneumoniae* [24], *Lactococcus lactis* [25], *Streptomyces lividans* [26] and *Lactobacillus casei* [27]. The gram-positive model organism *Bacillus subtilis* belongs to the closer relatives of *Listeria* and its maltodextrin/maltose utilization system has recently been characterized [28], [29]. Fermentation of maltose by *Listeria monocytogenes* has been described earlier [30], [31] but nothing was known so far about the mechanism of uptake and utilization of complex carbohydrates by *Listeria*. Here we show that *L. monocytogenes* EGD-e and other *Listeria* species contain genes for an ABC transporter and other essential functions involved in the efficient utilization of maltodextrin/maltose and describe their regulation by different carbohydrates.

## Results

### *Listeria monocytogenes* EGD-e contains a gene cluster with high similarity to the maltodextrin utilization system of *Bacillus subtilis*

**Figure 1:**
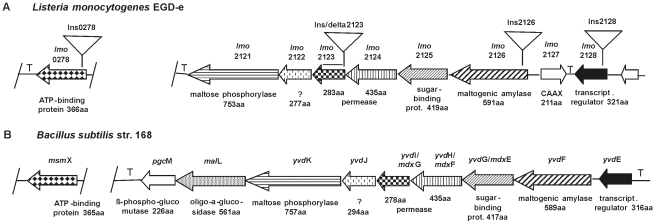
Schematic representation of maltodextrin utilization clusters. (A) from *L. monocytogenes* EGD-e as derived from the genome sequence [33] (genolist.pasteur.fr/ListiList); (B) the corresponding cluster from *B. subtilis* str. 168, according to [29] (genolist.pasteur.fr/SubtiList). The initial visualization of the genomic structures was done using the GECO utility [63]. Arrows are drawn to scale, genes putatively encoding proteins with similar functions have the same graphical pattern. Gene names are indicated above the arrows, experimentally proven or presumptive protein functions below, the protein lengths are also indicated. Triangles above the *L. monoyctogenes* cluster symbolize the position of insertion mutations and of the deletion generated in *lmo2123*. T  =  putative transcriptional terminator. The GenBank accession nos. for the respective genome sequences are AL591824 (*L.m*.) and AL009126 (*B.s*.).

The uptake and utilization of maltose and maltodextrin by *Bacillus subtilis* has been characterized in detail [28], [29], [32]. In this microorganism maltose is taken up via MalP, the maltose-specific EIICB component of a phosphotransferase (PTS) system, the phospho-α-glucosidase MalA is involved in maltose utilization. *MalP* and *malA* are under the transcriptional control of GlvR, the three genes are grouped together on the chromosome. Maltodextrins are transported by an ABC transporter consisting of the extracellular sugar-binding protein YvdH/MdxE and the membrane-bound permease subunits YvdH/MdxF and YvdJ/MdxG. The genes for these uptake proteins form a contiguous chromosomal cluster together with *yvdF* (maltogenic amylase), *yvdK* (maltose phosphorylase), *malL* (oligo-α-glucosidase) and *pgcM* (β-phospho-glucomutase). No function could be assigned to the product of *yvdJ*. MsmX, the gene of which is located outside of the cluster, has been identified as the cognate ATP-binding protein of the ABC transporter [29]. Fig. 1 (b) shows a schematic representation of the cluster. It has been proposed that YvdE is the respective transcriptional regulator, but this has not been proven experimentally. Recently it has been show that the maltogenic amylase YvdF is required for the generation of maltose from maltodextrins, which in turn are degradation products of extracellular starch or intracellular glycogen, respectively [28]. Furthermore it has been suggested that MalL and YvdK function in the degradation of maltose to glucose and glucose-1-phosphate, the latter one being converted to glucose-6-phosphate by PgcM. In *B. subtilis* maltose is exclusively taken up via the MalP phosphotransferase system, the YvdH/MdxE sugar-binding protein of the ABC transporter having a very low affinity for maltose [29].

A homology search for orthologues of the maltose PTS uptake and utilization proteins MalP, MalA and GlvR in the genome of *Listeria monocytogenes* EGD-e [33], using the BLAST [34] utility at the ListiList website (genolist.pasteur.fr/ListiList) gave the following results. Lmo1255, annotated as trehalose-specific enzyme IIBC, showed 22 percent identity/39 percent similarity to MalP of *B. subtilis* and Lmo2766, a RpiR family regulator, was found to be 22 percent identical/45 percent similar to GlvR. The best similarity (31 percent identical, 52 percent similar amino acids) to MalA was found for Lmo0521, annotated as phospho-β-glucosidase. Neither these three *L. monocytogenes* proteins nor other ones with lower similarity to the *B. subtilis* query proteins are encoded by clustered genes, as is the case in *B. subtilis*.

According to the ABCdb online resource for ABC transporter repertoires [35; www.abc-db.biotoul.fr] the *L. monocytogenes* EGD-e genome encodes for 57 complete or incomplete ABC transporters, i.e. comprising genes for at least one membrane-spanning domain (MSD) plus one for a nucleotide-binding domain (NBD) or for a MSD plus a substrate-binding protein (SBP), physically linked on the chromosome. Further, there are three “orphan” genes for NBDs. Among the 57 ABC transporters listed only Lmo2123-Lmo2125 were classified as similar to a maltodextrin ABC transporter. ListiList also showed that in *L. monocytogenes* EGD-e, *lmo2123*–*lmo2125* are part of a cluster of eight genes which, according to annotated gene product function and protein length, is almost identical to the major part of the maltodextrin utilization cluster of *B. subtilis*, this is schematically shown in figure 1.

A comparison by BLAST of the amino acid sequences derived from the cluster genes in *L. monoyctogenes* EGD-e and *B. subtilis* str. 168 (genolist.pasteur.fr/SubtiList) [36] showed that most of them had a significant homology (57–80 percent identity/70–92 percent similarity). Only Lmo2122 showed a low 25 percent identity/47 percent similarity to YvdJ of *B. subtilis*. As mentioned, the role of YvdJ is unknown, by conserved domain analysis [37] YvdJ as well as Lmo2122 were classified as integral membrane proteins of unknown function. One important difference between the two gene clusters is that in *B. subtilis* the gene for the putative transcriptional regulator YvdE is immediately upstream of the first gene (*ydv*F) of the utilization cluster, however, the transcriptional organization of this cluster is not yet known. In *L. monocytogenes* the putative regulator gene *lmo2128* is separated from the *yvd*F orthologue *lmo2126* by *lmo2127*, which is transcribed in the opposite direction. The gene product of *lmo2127* has been annotated as a type II CAAX prenyl endopeptidase. These poorly characterized enzymes are putative metal-dependent proteases [38], the function of Lmo2127 in *L. monocytogenes* is unknown.

Another obvious difference between *L. monocytogenes* and *B. subtilis* is that in the former bacterium no proteins with functions similar to MalL and PgmC could be found encoded downstream of the maltose phosphorylase gene. BLAST searches in ListiList with the *B. subtilis* proteins as query sequences yielded two oligo-1,6-glucosidases and four putative phosphoglucomutases. In all cases the similarities to the *B. subtilis* proteins were rather low and the genes were scattered.

In *B. subtilis* MsmX has been identified as the cognate ATP-binding protein for the MdxF/MdxG permease [29]. Lmo0278 of *L. monocytogenes* showed 72 percent identity/83 percent similarity to MsmX and therefore most probably is its equivalent.

### All sequenced *L. monocytogenes* strains and *Listeria* species contain genes which are identical or very similar to the *L. monocytogenes* EGD-e maltodextrin utilization genes

Homology searches in the *Listeria* genome databases Listilist (for *L. monocytogenes* and *L. innocua*) and LivaList (genolist.pasteur.fr/LivaList) for *L. ivanovii* and in the NCBI microbial genome database (http://www.ncbi.nlm.nih.gov/sutils/genom_table.cgi) yielded the following results. All *L. monocytogenes* isolates as well as *L. innocua* and *L. welshimeri* encode proteins which are virtually identical (90–100 percent identical/95–100 percent similar amino acids) to Lmo2121–Lmo2128 and Lmo0278. For *L. ivanovii* the homology slightly drops to 77–98 percent identity/83–99 percent similarity. In the case of *L. grayi* the homology was found to be significantly lower in most cases, with Lmo0278 showing the maximum value of 88 percent identity/94 percent similarity, Lmo2121/Lmo2122 exhibiting minimal 24–25 percent identity/44–50 percent similarity and the other proteins having 54–76 percent identity/68–88 percent similarity. This is in agreement with the established phylogenetic tree of *Listeria* which classifies *L. monocytogenes* and *L. innocua* as belonging to one group and *L. grayi* as the most distantly related one to all other *Listeria* species [39].

### Mutations in genes for the ABC transporter and for a putative transcriptional regulator abolish the growth of *L. monocytogenes* EGD-e on maltose or maltodextrin

Insertion mutations of the genes putatively encoding a subunit of the maltodextrin permease (*lmo2123*), the maltogenic amylase (*lmo2126*), the transcriptional regulator (*lmo2128*) and the ATP-binding protein (*lmo0278*) were constructed (see methods section). For *lmo2123* also a deletion mutant was constructed. The positions of the insertions and of the deletion are indicated in figure 1A by triangles (Ins*2123*, Ins*2126*, Ins*2128* and Ins*0278*) or a horizontal bar (Δ*2123*), respectively, above the gene symbols. The insertions into the monocistronic genes *lmo0278* and *lmo2128*, as well as the in frame deletion in *lmo2123* should have no polar effect of the transcription of other genes, whereas the insertions into *lmo2123* and *lmo2126* are supposed to abolish also the expression of the downstream genes in the transcription unit. It has recently been shown by others that *lmo2121*–*lmo2126* indeed constitute one operon [40].

**Figure 2:**
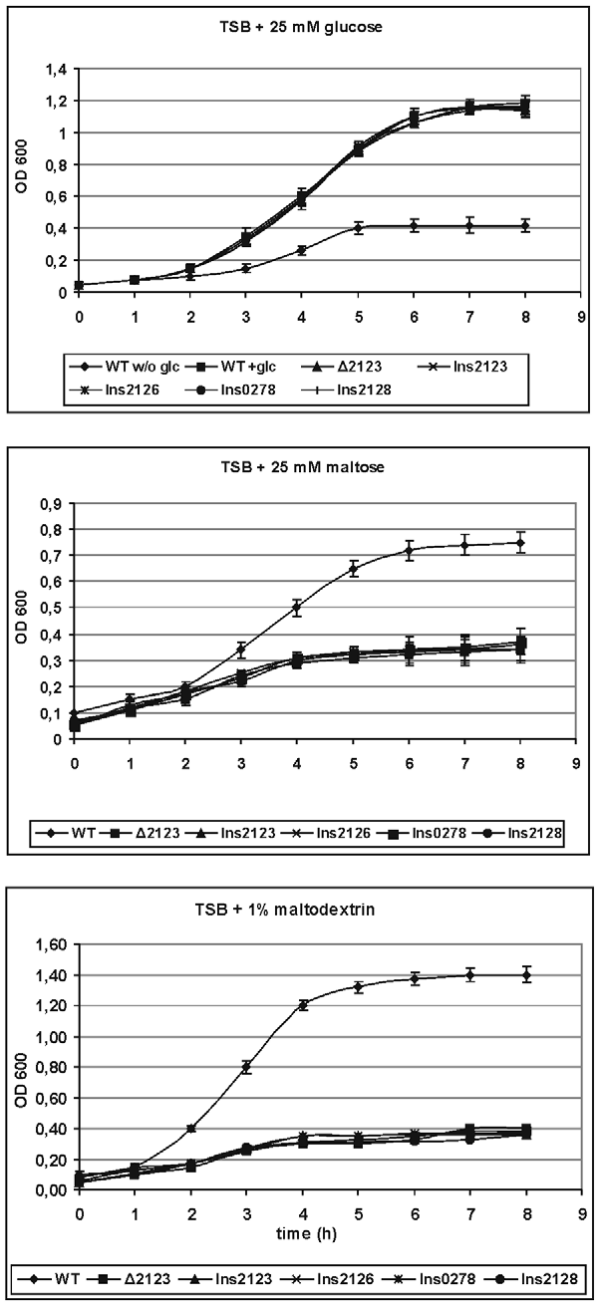
Growth curves in TSB. *L. monocytogenes* EGD-e wildtype and its isogenic mutants in TSB (tryptic soy broth, without glucose), supplemented with different sugars as indicated in the figure, at 37°C. For further explanations see text. The graph shows the means and standard deviations from three independent experiments.

Figure 2 (upper panel) shows that all strains showed good and identical growth in TSB with 25 mM glucose, demonstrating that the mutations did not cause a general growth defect. The wild type grew slowly and reached a final optical density of 0.4 only in TSB without sugar. The addition of 25 mM maltose resulted in a good growth of the wild type bacteria, but significantly less than in glucose-containing TSB. All mutants grew slowly and with a kinetics and final density similar to the wild type grown in sugar-free medium (Figure 2, middle panel), demonstrating that all were unable to utilize maltose to a measurable extent. Similar results were obtained in TSB with 1.0 percent maltodextrin (Figure 2, lower panel).

These results show that sustained growth of *L. monocytogenes* EGD-e in TSB is dependent on the addition and uptake of the supplemented carbon source and hence TSB is a suitable medium for such kind of investigations. Initial experiments with the defined medium HTM [41] did not yield sufficient cell mass as also previously shown for the defined medium MWB [31].

In order to verify that the growth defects observed for the insertion mutants in maltose-maltodextrin-containing TSB were only due to the insertion and not the result of secondary genetic alterations, revertants with a precise excision of the plasmid insert were isolated. Growth tests of the revertants in media containing maltose or maltodextrin confirmed that they grew like wild type (Table S1).

When multiplying in the cytosol of eukaryotic host cells *L. monocytogenes* can utilize different carbon sources, apart from glucose or glucose-6-phosphate derived from the glucose metabolism of the host, e.g. glycerol. The enzymatic breakdown of glycogen stores by host cell glycogen phosphorylase yields glucose-1-phosphate which also can be taken up by the bacteria [42], [43]. Maltose and maltodextrin are not taken up by mammalian cells and may potentially be generated by hydrolysis of glycogen under very unusual conditions only, e.g. upon release of lysosomal amylase into the cytoplasm. Therefore we did not expect an effect of the mutations in the maltose-maltodextrin utilization system on the multiplication of *L. monocytogenes* within Caco-2 enterocytes, which are known to contain rather large amounts of glycogen. This assumption was confirmed in infection experiments where the mutants Ins*2123* and Ins*2126* showed wild type multiplication in Caco-2 cells (Figure S1). Further we found that in wild type bacteria all genes within the cluster *lmo2121–lmo2128* were virtually not transcribed in Caco-2 cells (Figure S1), corroborating that these genes have no role during the intracellular phase of a *Listeria* infection.

### The transcription of the maltose-maltodextrin utilization gene cluster is induced by maltose and maltotriose and depends on the regulator Lmo2128

It has been shown that the expression of the *B. subtilis* maltodextrin/maltose utilization enzymes was induced by maltose or maltodextrin [29]. We measured by quantitative RT-PCR the transcription of *lmo2121* to *lmo2126* and *lmo2128* (putative regulator) after growth at 37°C into mid-log phase in TSB either containing no supplemented sugar or supplemented with glucose plus maltose, with maltose or maltotriose respectively. In parallel, the transcription of these genes was determined in the mutant Ins*2128* in which the putative transcriptional regulator of the gene cluster was inactivated by plasmid insertion.

**Figure 3:**
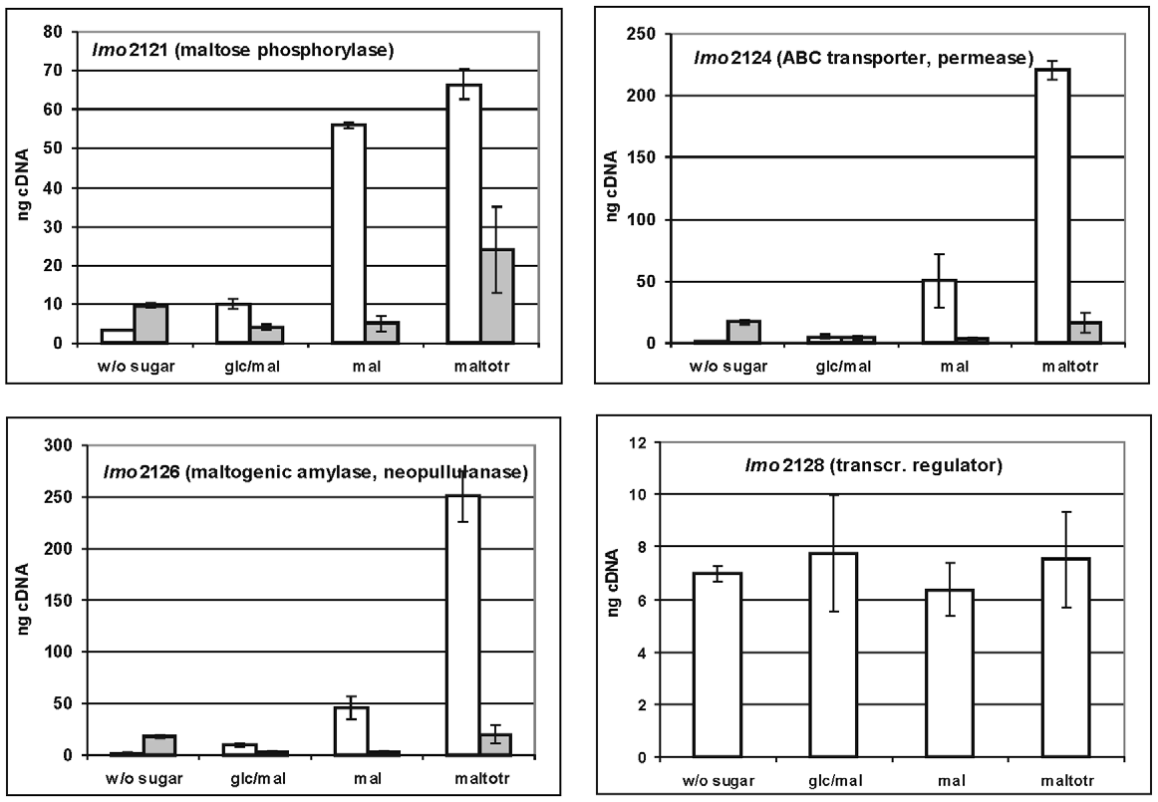
Role of Lmo2128 in regulation. Transcription analysis by qRT-PCR for *lmo2121*, *lmo2124*, *lmo2126* and *lmo2128* in wild type (open bars) and in the Ins*2128* mutant (grey bars). The strains were grown at 37°C in TSB without sugar or with 25 mM glucose+maltose (glc/mal), 25 mM maltose (mal) or 12.5 mM maltotriose (maltotr). Cells were harvested in mid-log phase (OD600 0.5–0.6). The results from the qRT-PCR analysis, obtained with an Opticon DNA Engine system (MJ Research Inc.) were normalized using *rpoB* as an internal standard [45], [62] and expressed as ng cDNA with an external standard as a reference, using the Opticon Monitor software (MJ Research Inc.). Means and standard deviations from three independent experiments.

The transcription of the representative genes *lmo2121*, *lmo2124* and *lmo2126* was massively induced upon the addition of maltose or maltotriose when compared to sugar-free medium (Figure 3). The gene for the putative transcriptional regulator Lmo2128 was transcribed constitutively. In medium containing both glucose and maltose this induction was completely abolished. In the mutant Ins*2128*, lacking the putative regulator, the induction also was abolished. The similar results for the other cluster genes *lmo2122*, *lmo2123* and *lmo2125* are shown in Figure S2. These results indicated that the expression of *lmo2121*–*lmo2126*, but not of *lmo2128* is subject to glucose repression and that Lmo2128 activates transcription of the entire cluster dependent on the presence of maltose or maltotriose in the medium. A potential binding site for Lmo2128, belonging to the LacI-GalR class of transcription regulators [44] could not be identified in the upstream sequences of the genes regulated by Lmo2128.

### Glucose repression of the maltose-maltodextrin utilization genes is independent of CcpA and is relieved in an Ins*hprK/P*-mutant

**Figure 4:**
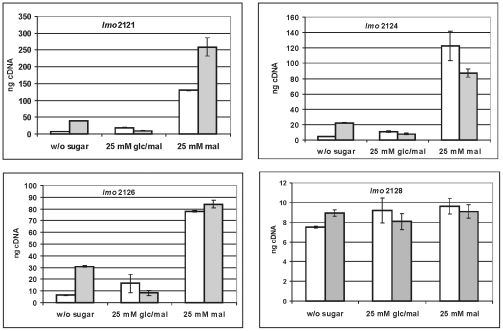
Role of CcpA in repression. Transcription analysis by qRT-PCR of *lmo2121*, *lmo2124*, *lmo2126* and *lmo2128* in wild type (open bars) and in a Ins*ccpA* mutant (grey bars). The strains were grown at 37°C in TSB without sugar or with 25 mM glucose+maltose (glc/mal) or 25 mM maltose (mal). Cells were harvested in mid-log phase (OD600 0.5–0.6). The results from the qRT-PCR analysis, obtained with a Opticon DNA Engine system (MJ Research Inc.) were normalized using *rpoB* as an internal standard [45], [62] and expressed as ng cDNA with an external standard as a reference, using the Opticon Monitor software (MJ Research Inc.). Means and standard deviations from three independent experiments.

The potential role of the catabolite control protein CcpA and of HprK/P in the transcriptional repression by glucose was assessed using appropriate mutants. The transcriptional repression by glucose of the representative genes *lmo2121*, *lmo2124* and *lmo2126* was not relieved in a mutant lacking CcpA (Figure 4). The similar results for the other cluster genes *lmo2122*, *lmo2123* and *lmo2125* are shown in Figure S3. As expected from the previous experiment, the mutation in *ccpA* also had no effect on the transcription of the regulator gene *lmo2128*.

**Figure 5:**
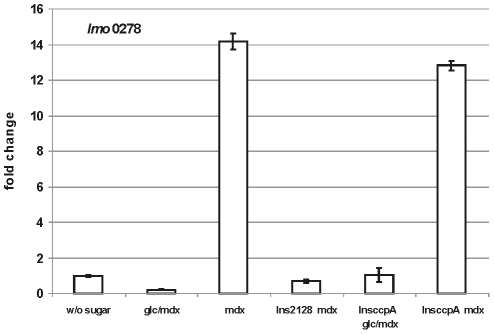
Transcription profile of *lmo0278*. Results of qRT-PCR for *lmo0278* in wild type and the Ins*2128* or Ins*ccpA* mutants. The strain was grown at 37°C in TSB without sugar or with 25 mM glucose + 1.0 percent maltodextrin (glc/mdx) or 1.0 percent maltodextrin (mdx). Cells were harvested in mid-log phase (OD600 0.5–0.6). The results from the qRT-PCR analysis, obtained with a StepOnePlus Real-Time PCR system (Applied Biosystems Inc.) were normalized using *rpoB* as an internal standard [45], [62] and expressed as fold change with the values for wild type without sugar (WT w/o sugar) set as 1.0. Calculations were performed with the StepOne Software v2.1 (Applied Biosystems Inc.). Means and standard deviations from three independent experiments.

**Figure 6:**
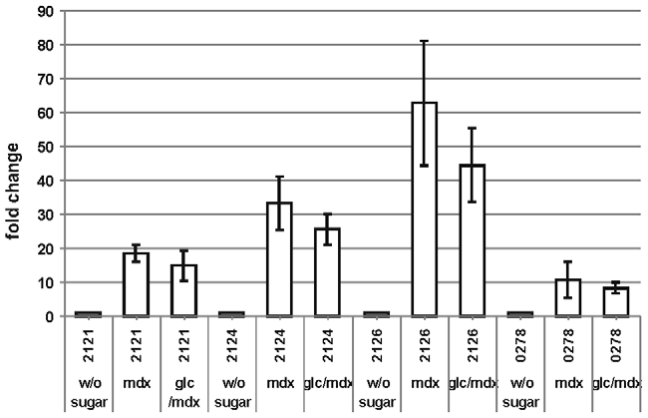
Role of HprK in repression. Transcription analysis by qRT-PCR of *lmo2121*, *lmo2124*, lmo*2126* and *lmo0278* in the Ins*hprK* mutant. The strain was grown at 37°C in TSB without sugar or or 1.0 percent maltodextrin (mdx) or with 25 mM glucose + 1.0 percent maltodextrin (glc/mdx). Cells were harvested in mid-log phase (OD600 0.5–0.6). The results from the qRT-PCR analysis, obtained with a StepOnePlus Real-Time PCR system (Applied Biosystems Inc.) were normalized using *rpo*B as an internal standard [45], [62] and expressed as fold change with the values for wild type without sugar (WT w/o sugar) set as 1.0. Calculations were performed with the StepOne Software v2.1 (Applied Biosystems Inc.). Means and standard deviations from three independent experiments.

The gene *lmo0278* for the ATP-binding protein of the ABC transporter had the same transcription profile as the cluster genes (Figure 5). It was repressed by glucose, induced by maltodextrin, not expressed in the Ins*212*8 regulator mutant and inactivation of *ccpA* did not relieve its glucose repression. Further, the transcription of several representative genes of the maltose-maltodextrin utilization cluster was measured in an Ins*hprK/P*-mutant of *L. monocytogenes* [45]. The repression by glucose of *lmo2121*, *lmo2124*, *lmo2126* and *lmo0278* was abolished in the mutant (Figure 6). It can be anticipated that the other genes in the operon (*lmo2122*, *lmo2123* and *lmo2125*) are regulated in the same way.

## Discussion

It has previously been suggested [46] that in *L. monocytogenes* EGD-e maltose is taken up and utilized by the proteins encoded by *lmo0858*–*lmo0865*. This assumption was based on a comparison of the genome organization of *Lactococcus lactis*, *Lactobacillus plantarum* and *Listeria monocytogenes.* Lmo0859–Lmo0861 are annotated in both ListiList and the ABCdb database of ABC transporters [35] as components of a sugar ABC transporter without a particular specificity, hence they were indeed suitable candidates for the uptake of maltose. However, our results clearly show that in fact maltose is taken up by *L. monocytogenes* EGD-e via the Lmo2121–Lmo2124 and Lmo0278 ABC transporter, which is under the transcriptional control of Lmo2128. In *B. subtilis* maltose is solely transported by the MalP enzyme IICB protein, this has been attributed to the very low affinity for maltose of the YvdG/MdxE substrate-binding protein of the ABC transporter preventing an efficient transport of maltose [29]. In contrast, our growth experiments with wild type and mutants showed that *L. monocytogenes*, which lacks a MalP orthologue, obviously can take up maltose by the ABC transporter. Its sugar-binding protein Lmo2125 has 63 percent identity/77 percent similarity to the *B. subtilis* protein, the observed differences between the two proteins seem to cause a sufficient affinity for maltose in the case of *L. monocytogenes*. Furthermore, we show for the first time that *L. monocytogenes* can also utilize maltodextrins, using the Lmo2121–Lmo2126 and Lmo0278 system for uptake and metabolism.

As *B. subtilis*, the *L. monocytogenes* gene cluster encodes an enzyme (Lmo2126) which has been annotated as maltogenic amylase. For *B. subtilis* it has been shown that this enzyme, YvdF/MAase [28] hydrolyses maltodextrins to maltose. In the case of *B. subtilis* the maltodextrins are derived either from glycogen stores, degraded by the sequential action of the glycogen phosphorylase GlgP and the pullulanase AmyX, or from extracellular starch, hydrolyzed by the secreted amylase AmyE [28]. *L. monocytogenes* lacks the enzymes for glycogen biosynthesis and metabolism. It also has no gene encoding a secreted amylase and we could not detect any amylolytic activity in cell extracts or in culture supernatants (data not shown). Also a previous analysis of the proteins secreted by *L. monocytogenes* did not detect Lmo2126 (or any other potentially starch-degrading enzyme) in the culture supernatant [47]. However, on decaying vegetation in soil *L. monocytogenes* most probably will be associated with other microorganisms able to hydrolyze starch. In the intestine, the pancreatic amylase will generate maltose and maltodextrins from nutrional starch. In both cases *L. monocytogenes* is independent of secreting an amylolytic enzyme by its own. So most presumably Lmo2126 has the same function as YvdF/MAase of *B. subtilis*, i.e. intracellular generation of maltose and maltotriose from longer maltodextrins.

**Figure 7:**
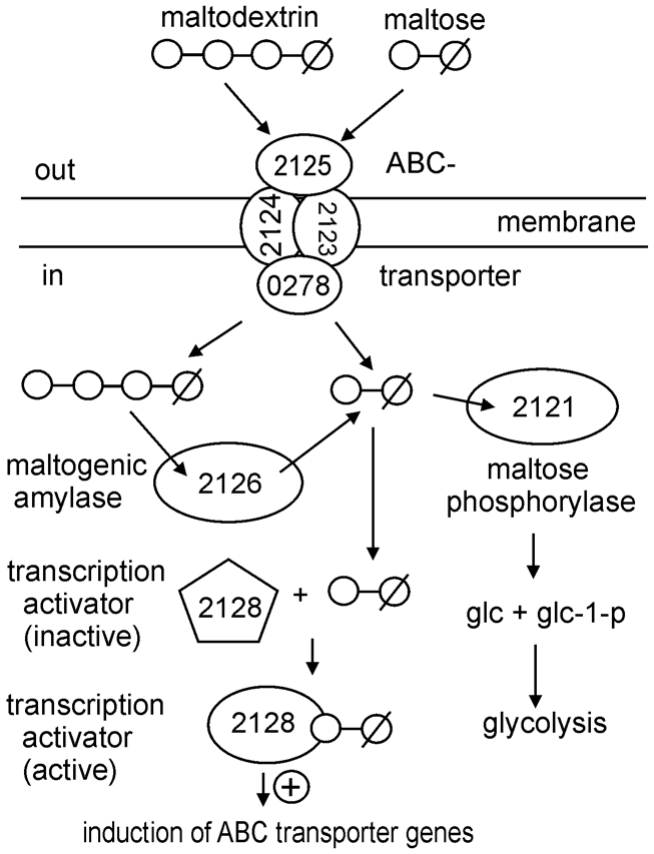
Hypothetical model of the ABC transporter system. The putative function and regulation of the *L. monocytogenes* ABC transporter and utilization system for maltose and maltodextrin, adapted from [28], [29], [49]. Explanations in the text.

It has been proposed that YvdE of *B. subtilis* is the cognate transcriptional regulator of the maltodextrin-utilization cluster [29], but so far there is no experimental evidence for this. Here we show that Lmo2128, the *L. monocytogenes* orthologue of YvdE, is a positive regulator of the transcription of *lmo2121*–*lmo2126* and of *lmo0278*, i.e. of all components of the maltose-maltodextrin utilization system. A similar positive regulatory effect has previously been shown for MalR of *L. lactis* [25]. Transcriptional activation by Lmo2128 was observed with maltose, maltotriose and higher maltodextrins (4–7 glucose moieties) in the growth medium. Our experiments suggest that maltose is the genuine intracellular inducer, with Lmo2126, the maltogenic amylase, generating maltose from maltodextrins in the cytoplasm. Domain analyses (data not shown) of Lmo2128 using SMART [48] and the Conserved Domain Database [37] showed that the protein belongs to the LacI/GalR family of transcriptional regulators. It has a N-terminal DNA-binding helix-turn-helix motif, followed by a PBP1 type sugar-binding domain (data not shown), a PRD (PTS regulation domain) [49], [50] was not found. A consensus sequence corresponding to a LacI-family operator motif [44] could not be detected in the 5′ upstream region of the genes regulated by Lmo2128, so the binding site for this regulator has yet to be identified. Figure 7 shows a hypothetical model for the function and regulation by Lmo2128 of the *L. monocytogenes* maltodextrin/maltose uptake and utilization system.

The transcription of all the genes of the system was repressed by glucose, i.e. it is affected by carbon catabolite repression (CCR). In gram-positive bacteria, this kind of repression is fundamentally different from the mechanisms operating in enterobacteria. It involves components of the phosphotransferase sugar uptake system and in most cases the catabolite control protein CcpA and its corepressors Hpr or Crh [49], [51], [52], [53]. A homologue of CcpA has been found in *L. monocytogenes* [54], Hpr is also present, Crh is missing in this bacterium (our own BLAST search, results not shown). A lack of CcpA did not abrogate the repression by glucose of *lmo2121*–*lmo2126* and *lmo0278*, also in the presence of the inducer in the external medium. CcpA-independent catabolite repression by glucose has been described for several genes of *B. subtilis* [55] and also for the virulence genes of *L. monocytogenes* [45], [54]. In the presence of glucose the Hpr protein, the common component of phosphotransferase (PTS) transport systems for various sugars, is phosphorylated at Ser-46, yielding P-Ser-Hpr. This phosphorylation is catalyzed by the Hpr kinase/phosphorylase HprK/P and P-Ser-Hpr in a complex with CcpA binds to target sites (*cre-*boxes) in the upstream regions of regulated genes. But P-Ser-Hpr may also directly bind and inhibit the activity of ATP-binding proteins of ABC transporters, thus preventing the uptake of inducing sugar, this has been designated as inducer exclusion. In case of the maltose transport in *Lactobacillus casei* and *Lactococcus lactis* good evidence has been obtained that glucose repression is mediated by such an inhibition of the maltose ABC transporter MalFGK [27], [49], [56]. As we could show here, the repression by glucose of the genes of the maltose-maltodextrin utilization cluster of *L. monocytogenes* EGD-e is also independent of CcpA but relieved in a HprK/P mutant. Therefore we tentatively propose inducer exclusion as the underlying mechanism. However, we cannot exclude that other, so far unknown mechanisms are operating here.

Concerning a potential role of maltose-maltodextrin utilization during a natural infection by *L. monocytogenes* it has been shown that environmental oligosaccharides are involved in the enterococcal biofilm formation and colonization of the gastrointestinal tract [57], which is also the site of entry of *L. monocytogenes*. For *Streptoccocus pyogenes*, a pathogen which colonizes the carbohydrate-rich mucosa of the oropharynx, a direct link between carbohydrate utilization and virulence has been demonstrated [58], [59]. Recently is has been shown that *lmo2121*–*lmo2126* are significantly up regulated in the intestinal lumen of orally infected mice [44]. Therefore it will be interesting to see in further studies if *L. monocytogenes* carrying mutations in the maltose-maltodextrin utilization system are impaired in their ability to colonize the intestine.

## Materials and Methods

### General techniques

PCR amplifications, cloning procedures, isolation of chromosomal DNA, and DNA manipulations were carried out according to standard procedures [60]. Cycle sequencing was performed using the CEQ Dye Terminator Cycle Sequencing Quick Start kit (Beckman Coulter, Fullerton, CA), and sequencing reactions were run on a XL2000 Beckman Coulter sequencer.

### Bacterial strains, plasmid, and cell line

*L. monocytogenes* Sv1/2a EGD-e (ATCC BAA-679) was obtained from T. Chakraborty (University of Giessen, Germany). Insertion mutants in *ccpA* and *hprK*, respectively, were constructed and provided by Mertins et al. [45]. E. *coli* strain TG1 and plasmid pG^+^host4 [61] were kindly provided by E. Maguin (INRA Jouy en Josas, France). Human colon epithelial cells (Caco-2 cells) were from the American Type Culture Collection (ATCC HTB-37) and were cultured at 37°C and 5% CO_2_ in RPMI 1640 (Gibco) supplemented with 10% heat-inactivated fetal calf serum (FCS) (Biochrom KG, Germany).

### Media and growth conditions

*L. monocytogenes* was grown in brain heart infusion (BHI) or tryptic soy broth without glucose (TSB; Sigma Co.) at 37°C. Glucose, maltose (analytical grade, purity >99 percent; less pure preparations may contain significant amounts of maltodextrin) and maltodextrin used as supplements were purchased from Sigma Co. For some growth test the defined medium HTM [41] was used. Cultivation of *E. coli* was in Luria-Bertani (LB) medium at 37°C. For transformation experiments media were supplemented with erythromycin to final concentrations of 300 µg ml^−1^ for *E. coli* or 5 µg ml^−1^ for *L. monocytogenes*. For growth tests of *L. monocytogenes*, 300 µl of an overnight culture were diluted into 10 ml prewarmed TSB or HTM and shaken at 190 rpm. The optical density of the cultures was measured every hour in a photometer (Ultrospec, AmershamBiosciences) at 600 nm in 1 cm cuvettes.

### Mutant construction

PCR-amplified fragments from the 5′ region of the respective genes were cloned into the temperature-sensitive integration vector pG^+^host4 and transformed into *E. coli* TG1 [61]. *L. monocytogenes* EGD-e was transformed with the plasmid constructs and plasmid integrants were selected at the non-permissive temperature of 42°C on erythromycin-containing BHI agar. To obtain revertants, the mutant strains were subcultured twice for 24 hrs, in BHI without antibiotics at 25°C. At this temperature the plasmid origin of replication was fully active which favored plasmid excision [61]. Serial dilutions of the subcultures were plated on BHI without antibiotic and erythromycin-sensitive clones were identified by replica-plating on erythromycin-containing medium, plasmid excision was confirmed by PCR. In addition, an in frame deletion mutant in *lmo2123* (Δ*2123*) was constructed by a similar approach, using a plasmid construct carrying both 5′ and 3′gene fragments and selection first at 42°C on erythromycin-containing medium for insertion and subsequently at 30°C on antibiotic-free medium for plasmid loss and deletion. The correct insertion/deletion was verified by PCR and DNA sequencing (data not shown). Oligonucleotides used for mutant construction are listed in table S2.

### RNA isolation

For *in vitro* experiments, *L. monocytogenes* was grown in TSB to an optical density of 1.0 at 600 nm corresponding to the late logarithmic phase. For *in vivo* assays 250-ml tissue culture flasks with confluent Caco-2 cells were infected with *L. monocytogenes* at an m.o.i. of 20 as described [42]. Cells were lysed 6 h post infection with cold distilled water. Mammalian cell debris was removed by centrifugation at 1,000×*g* for 10 min at 4°C, leaving only the bacteria in the supernatant. Bacteria were pelleted at 6,000×*g* for 10 min at 4°C. Total RNA was isolated using the RNeasy mini kit (Qiagen) according to the manufacturer's protocol with some modifications to lysis of the bacteria. Cell pellets were suspended in 700 µl RLT lysis buffer (Qiagen) and placed in a 2-ml tube, filled with Lysing matrix B (Q BIOgene). The tube was shaken three times for 45 s each time with a 1-minute interval on ice between each shaking at a speed setting of 6.5 in a bead beater FP120 FastPrep cell disrupter (Bio101 Savant). Residual DNA was removed on a column with RNase-free DNase (Qiagen).

### Real-time qRT-PCR

Real-time quantitative reverse transcriptase PCR (qRT-PCR) was conducted on total RNA isolated. The absence of DNA from RNA samples was verified by PCR prior to reverse transcription, using *rpoB*-specific primers. 5 µg of total RNA was reverse transcribed with random hexamers and SuperScript II™ Reverse Transcriptase (Invitrogen) according to the manufacturer's instruction. Real-time qRT-PCR in a final volume of 50 µl was carried out in an Opticon DNA Engine (MJ Research) or in a final volume of 25 µl in a StepOnePlus Real Time PCR System (Applied Biosystems) according to the manufacturer's protocols. The Absolute™ QPCR SYBR^R^ Green Mix (Thermo Scientific,UK) was used. Transcript analysis was done with the Opticon Monitor (MJ Research) or StepOne v2.1 (Applied Biosystems) software, respectively. The housekeeping gene *rpoB* served as an internal standard [45], [62]. All primers used are listed in Table S2.

## Supporting Information

Table S1 Growth of wild type and revertants in TSB supplemented with maltose or maltodextrin, respectively. Indicated is the optical density at 600 nm after 24 hrs. at 37°C, means from three independent experiments.
(0.01 MB PDF)

Table S2 Oligonucleotide primers used in the study.
(0.02 MB PDF)

Figure S1 (A) Intracellular multiplication in Caco-2 enterocytes of *L. monocytogenes* EGD-e wild type and its isogenic mutants *Ins2123* and *Ins2126*. For experimental details see methods section. Colony forming units per ml (c.f.u. ml-1) in the host cell lysate were determined after an initial 45 min. adhesion and invasion phase, this is designated t = 0, and 3, 6 and 24 hrs. later. (B) Transcription analysis by qRT-PCR of *lmo2121-2126* and *lmo2128* at t = 6 hrs. after infection of Caco-2 enterocytes with wild type *L. monocytogenes* EGD-e. The virulence genes *actA* and *plcA*, which are readily expressed within eukaryotic host cells, served as positive controls. Means and standard deviations from three independent experiments.
(0.09 MB TIF)

Figure S2 Transcription analysis by qRT-PCR of *lmo2122, lmo2123, lmo2125* in wild type (open bars) and in the *Ins2128* mutant (grey bars). The strains were grown at 37°C in TSB without sugar or with 25 mM glucose+maltose (glc/mal), 25 mM maltose or 12.5 mM maltotriose (maltotr). Cells were harvested in mid-log phase (OD600 0.5–0.6). Means and standard deviations from three independent experiments.
(0.13 MB TIF)

Figure S3 Transcription analysis by qRT-PCR of *lmo2122, lmo2123, lmo2125* in wild type (open bars) and in the *InsccpA* mutant (grey bars). The strains were grown at 37°C in TSB without sugar or with 25 mM glucose+maltose (glc/mal) or with 25 mM maltose. Cells were harvested in mid-log phase (OD600 0.5–0.6). Means and standard deviations from three independent experiments.
(0.12 MB TIF)
