# Supplementary material for: Maltose and Maltodextrin Utilization by Listeria monocytogenes Depend on an Inducible ABC Transporter which Is Repressed by Glucose
Source: PLoS One. 2010 Apr 27;5(4):e10349. doi: 10.1371/journal.pone.0010349 (PMC2860498; doi:10.1371/journal.pone.0010349)
Supplement: Table S1 — Growth of wild type and revertants in TSB supplemented with maltose or maltodextrin, respectively. Indicated is the optical density at 600 nm after 24 hrs. at 37°C, means from three independent experiments. (0.01 MB PDF) [file pone.0010349.s001.pdf]

Table S1. Growth of wild type and revertants in TSB supplemented with maltose or maltodextrin, respectively. Indicated is the optical density at 600 nm after 24 hrs. at 37 °C, means from three independent experiments.

Strain	TSB+25 mM maltose	TSB+ 1% maltodextrin
Wild type	0.85 ± 0.08	1.10 ± 0.10
Revertant Ins*2123*	0.84 ± 0.05	1.05 ± 0.11
Revertant Ins*2126*	0.85 ± 0.06	1.05 ± 0.09
Revertant Ins*2128*	0.86 ± 0.05	1.07 ± 0.10
Revertant Ins*0278*	0.86 ± 0.08	1.08 ± 0.11
