# Supplementary material for: Maltose and Maltodextrin Utilization by Listeria monocytogenes Depend on an Inducible ABC Transporter which Is Repressed by Glucose
Source: PLoS One. 2010 Apr 27;5(4):e10349. doi: 10.1371/journal.pone.0010349 (PMC2860498; doi:10.1371/journal.pone.0010349)
Supplement: Table S2 — Oligonucleotide primers used in the study. (0.02 MB PDF) [file pone.0010349.s002.pdf]

**Table S2.** Oligonucleotide primers used in the study.

| **Oligoname (target gene)** | **Sequence** |
|---|---|
| **Mutant construction** | Introduced restriction sites underlined |
| 2123 upx (XbaI) | 5-´TCCTTTCTAGACAAAAGTAGC- 3´ |
| 2123deE1 (EcoRI) | 5´-CCCAGAATTCTTAACGATTGC-3` |
| 2123dwxo (XhoI) | 5´-CAATGCTCGAGAAACTGCTC-3` |
| 2123 Up | 5´-CGATTAATTCACAAGTTATCC-3´ |
| 2123upE (EcoRI) | 5´-CTGATCTTTCATGAATTCTC-3´ |
| Amy B2-2126(BamHI) | 5´-AAAGCAGGGATCCACCATCAA-3´ |
| Amy x3-2126 (XhoI) | 5´-GAATACTCGAGTCTGTTGTAT -3´ |
| Amy up (2126) | 5´-AAGCTTGCCAGAATTTCCAG-3´ |
| 2128UP | 5´-TCGGACTTTTAGAGAGAATGC-3' |
| 2128XA (XbaI) | 5'-GGCGACTCTAGAAGATGTTG-3' |
| 2128XO (XhoI) | 5'-TTGTGTCTCGAGTTGCAAAG-3' |
| 0278-I (up) | 5´-ATAGAAAGAGGCGAAGAAAG-3´ |
| 0278-EcoRI | 5´-TGACAAAGAATTCATCGTTTTTG-3´ |
| 0278-BamHI | 5´-TTTTAAGGATCCAGTTAAGCC-3´ |
| **pG+host4** | Flanking multiple cloning site |
| Blue R | 5´-GGAAACAGCTATGACCATC-3´ |
| M13 | 5´-GTAAAACGCGGCCAGT-3´ |
| **Primers for qRT-PCR** | |
| 2121R | 5´-GATTTTCTGCGCGTTAATTTTC-3' |
| 2121FI | 5'-ATTGGCCCTAAAGGATTTACTG-3' |
| 2122R | 5'-TCATCCAAATGAAAAGTGTCGT-3' |
| 2122FI | 5'-CTACCTTAAGCGAAATAACGTC-3' |
| 2123R | 5'-AAGTCCTACCGCAATCGTTAAG-3' |
| 2123FI | 5'-ACTTGATGGCGCAGGACATTTC-3' |
| 2124R | 5'-TCCAAAAAGACGTTTCCCTTTA-3' |

| | |
|---|---|
| 2124Fl | 5’-TGGGTTGGGTTTAAGAACTTC-3’ |
| 2125R | 5’-ACATTAAGTTTTCGGCACCTGT-3’ |
| 2125Fl | 5’-ACAAATGAAGTACCGGCTAAC-3’ |
| 2126R | 5’-CCGTCAATATCAAACTCACGAA-3’ |
| 2126Fl | 5’-GTTTCCCAGTTCGTCAAAATG-3’ |
| 2128Fl | 5’-ATTGCGAAATAGTTCCAGTATC-3’ |
| 2128R | 5’-TTTTGGCGATGAAATCCTATCT-3’ |
| 0278R | 5´-GCCAAGGATATTTGCTGCAT-3´ |
| 0278F | 5´-CTTTGCGTATGATTGCTGGA-3´ |
| plcAR | 5’-CCGCGGACATCTTTTAATGT-3’ |
| plcAFl | 5’-CGAGCAAAACAGCAACGATA-3’ |
| actAR | 5’-AAGCATCCGCAACTGACTCT-3’ |
| actAFl | 5’-ATAGCGCAGCGGAAATTAAA-3’ |
| rpoBqRT-F | 5’-CACCCTGAAGCTCCATTTGT-3’ |
| rpoBqRT-R | 5’-ACACGACGAACCCAGATTTC-3’ |
