# Supplementary figures and images for: Maltose and Maltodextrin Utilization by Listeria monocytogenes Depend on an Inducible ABC Transporter which Is Repressed by Glucose
Source: PLoS One. 2010 Apr 27;5(4):e10349. doi: 10.1371/journal.pone.0010349 (PMC2860498; doi:10.1371/journal.pone.0010349)

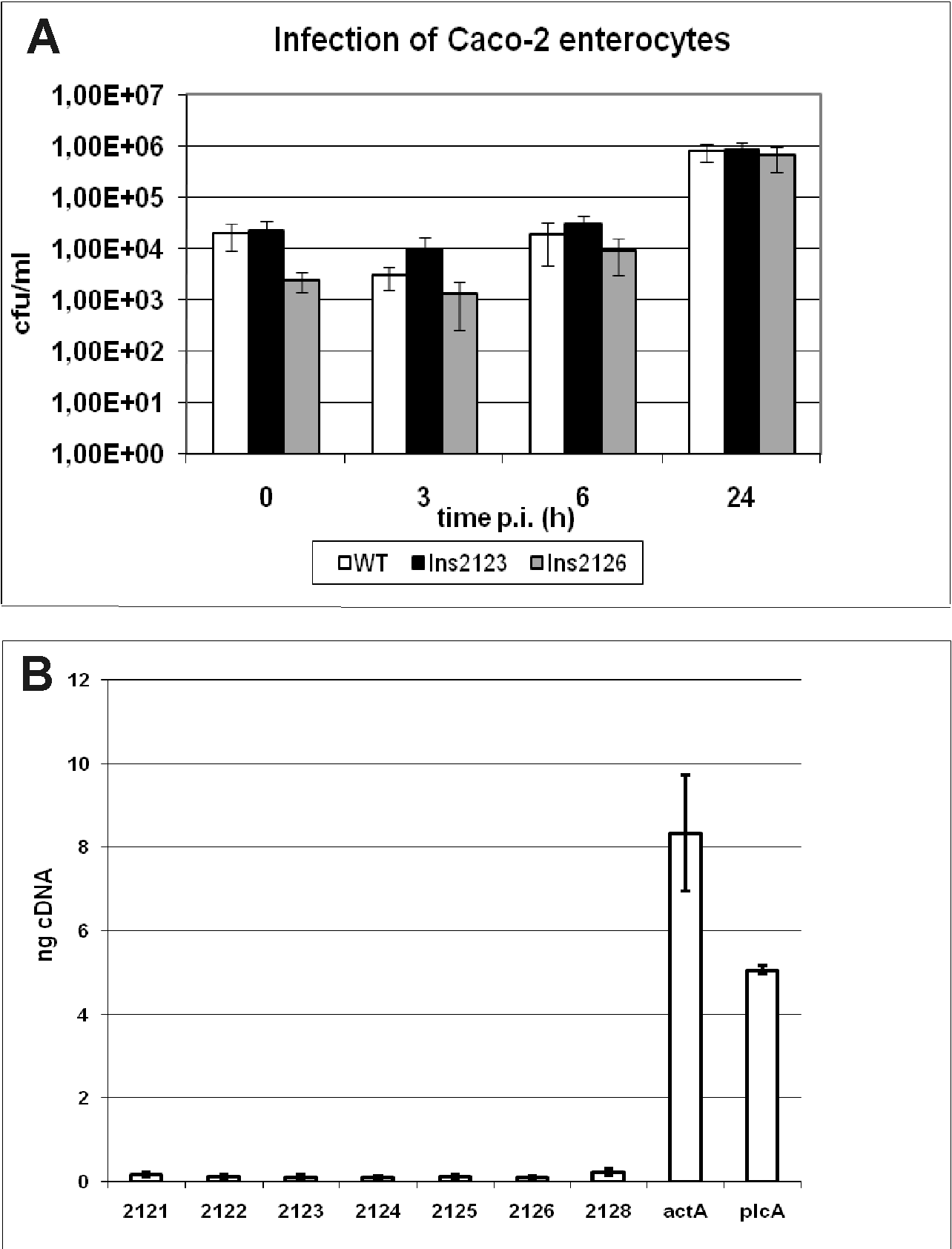

Supplement: Figure S1 — (A) Intracellular multiplication in Caco-2 enterocytes of L. monocytogenes EGD-e wild type and its isogenic mutants Ins2123 and Ins2126. For experimental details see methods section. Colony forming units per ml (c.f.u. ml-1) in the host cell lysate were determined after an initial 45 min. adhesion and invasion phase, this is designated t = 0, and 3, 6 and 24 hrs. later. (B) Transcription analysis by qRT-PCR of lmo2121-2126 and lmo2128 at t = 6 hrs. after infection of Caco-2 enterocytes with wild type L. monocytogenes EGD-e. The virulence genes actA and plcA, which are readily expressed within eukaryotic host cells, served as positive controls. Means and standard deviations from three independent experiments. (0.09 MB TIF) [file pone.0010349.s003.tif]

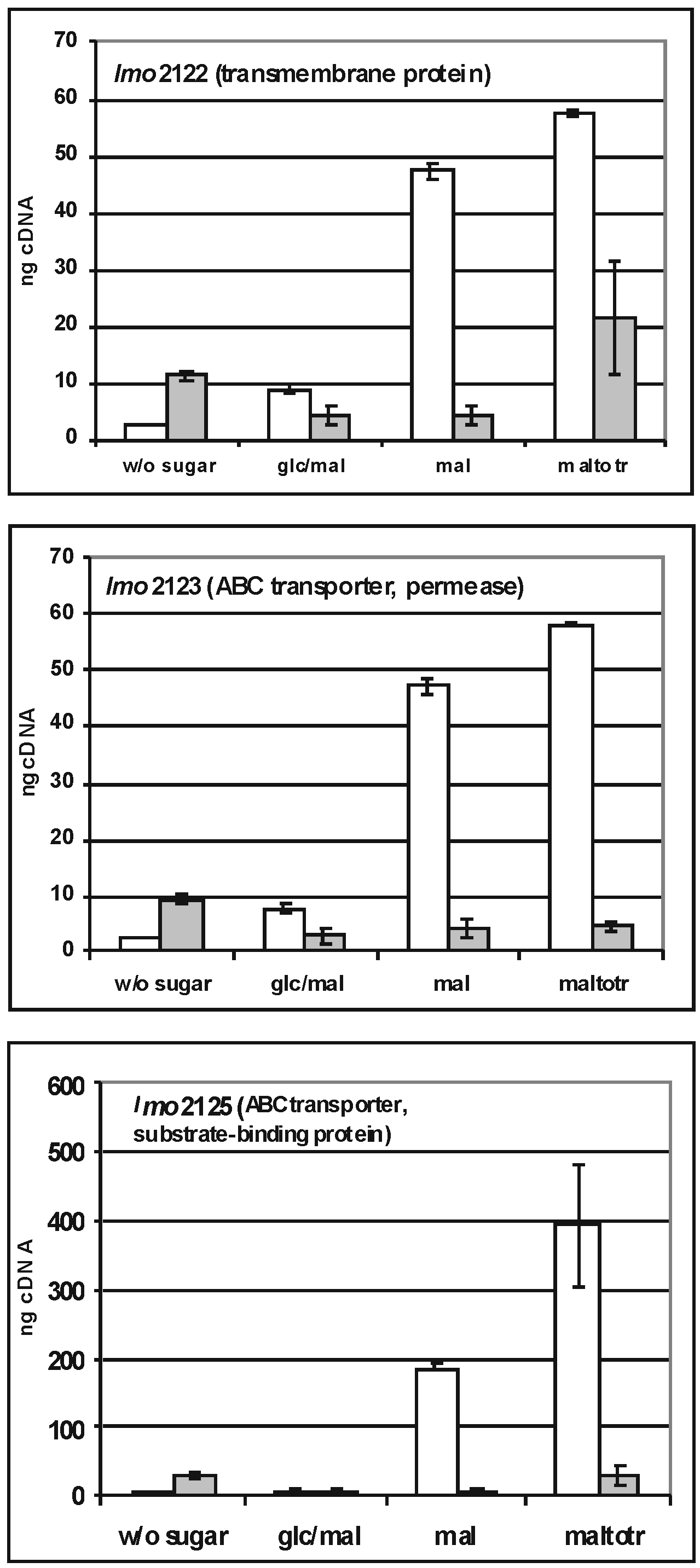

Supplement: Figure S2 — Transcription analysis by qRT-PCR of lmo2122, lmo2123, lmo2125 in wild type (open bars) and in the Ins2128 mutant (grey bars). The strains were grown at 37°C in TSB without sugar or with 25 mM glucose+maltose (glc/mal), 25 mM maltose or 12.5 mM maltotriose (maltotr). Cells were harvested in mid-log phase (OD600 0.5–0.6). Means and standard deviations from three independent experiments. (0.13 MB TIF) [file pone.0010349.s004.tif]

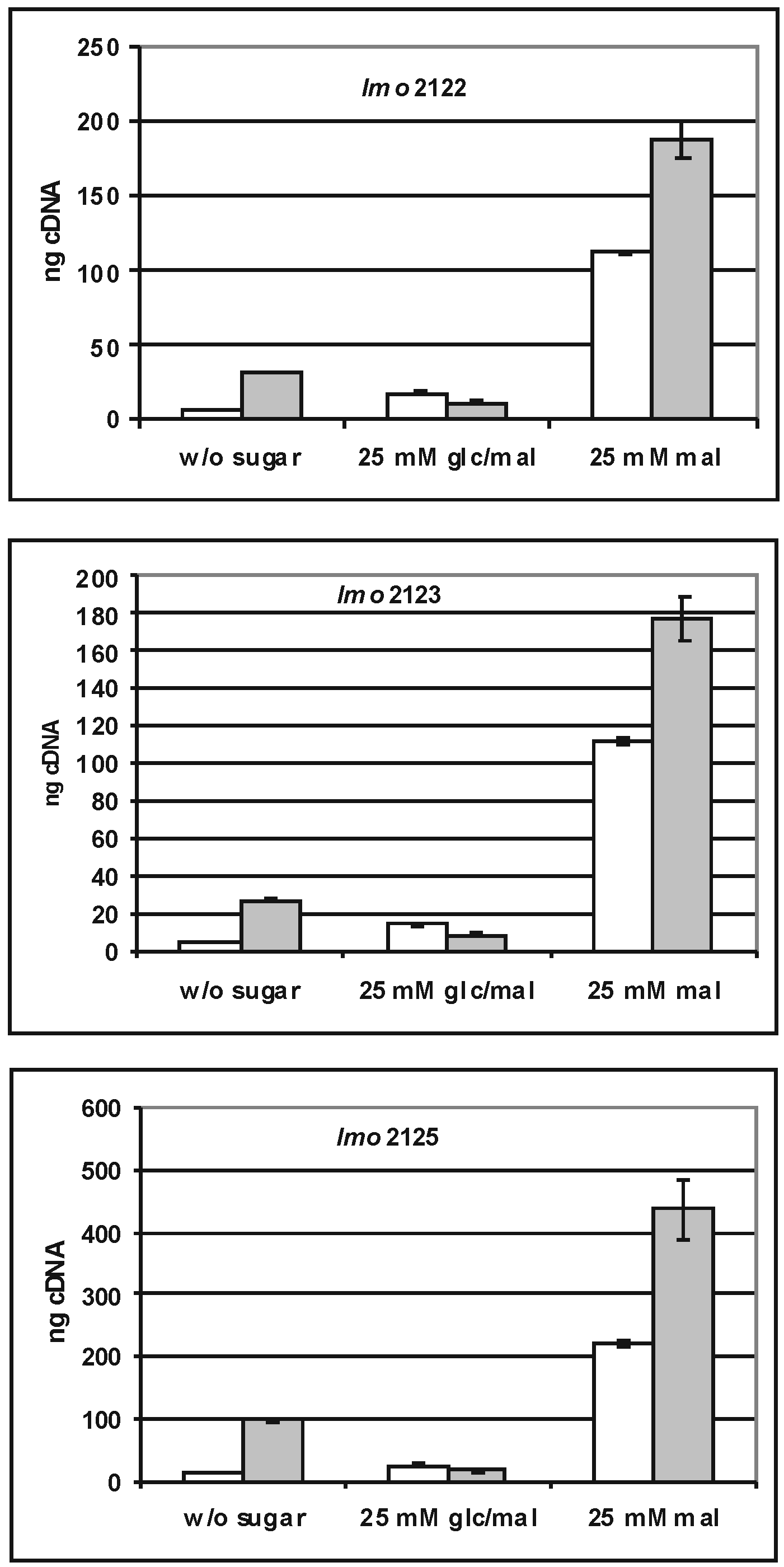

Supplement: Figure S3 — Transcription analysis by qRT-PCR of lmo2122, lmo2123, lmo2125 in wild type (open bars) and in the InsccpA mutant (grey bars). The strains were grown at 37°C in TSB without sugar or with 25 mM glucose+maltose (glc/mal) or with 25 mM maltose. Cells were harvested in mid-log phase (OD600 0.5–0.6). Means and standard deviations from three independent experiments. (0.12 MB TIF) [file pone.0010349.s005.tif]
